# Detection of pathogens and antimicrobial resistance genes directly from urine samples in patients suspected of urinary tract infection by metagenomics nanopore sequencing: A large‐scale multi‐centre study

**DOI:** 10.1002/ctm2.824

**Published:** 2023-04-26

**Authors:** Manjiao Liu, Simin Yang, Susheng Wu, Li Chen, Shan Li, Zhenzhong Li, Mingzhe Zhou, Lili Wang, Hui Xu, Ryon Liu, Yi Fang, Weichun Huang, Min Zhang, Wenzheng Guo, Yan Dai, Yong Ren, Hao Guo, Wenjuan Wu

**Affiliations:** ^1^ Department of Laboratory Medicine, Shanghai East Hospital Tongji University School of Medicine Shanghai P. R. China; ^2^ State Key Laboratory of Translational Medicine and Innovative Drug Development Jiangsu Simcere Diagnostics Co., Ltd. Nanjing P. R. China; ^3^ Nanjing Simcere Medical Laboratory Science Co., Ltd. Nanjing P. R. China; ^4^ State Key Laboratory of Genetic Engineering, School of Life Science Fudan University Shanghai P. R. China; ^5^ Department of Laboratory Medicine Huadong Hospital, Fudan University Shanghai P. R. China; ^6^ Department of Laboratory Medicine Shanghai Children's Medical Center Shanghai Jiao Tong University School of Medicine Shanghai P. R. China

Dear Editor,

Previous studies using metagenomics nanopore sequencing to detect pathogens and/or antimicrobial resistant (AMR) genes for urinary tract infection (UTI) had small sample sizes and only focused on UTI caused by bacteria.^1^, ^2^ Besides, the bioinformatics pipeline was not optimized. In this study, we optimized the metagenomics nanopore sequencing and bioinformatics pipeline suitable for detecting pathogens and AMR genes from urine samples. We enrolled 1045 samples from four hospitals to assess the pathogen detection, UTI diagnosis and AMR gene detection performance of the pipeline (Figure S1, Tables S1 and S2).

We developed the wet‐lab pipeline by adjusting the amount of saponin and the PCR system based on a previously published pipeline.^3^ The limits of detections for three common UTI pathogens were determined using spike‐in samples, and precision test results confirmed the stability and repeatability of the methods (Table S3).

To accurately detect pathogens and filter out false‐positive results, a multi‐step workflow using parameters, including blast cut‐off, second best match reads ratio, minimal reads and RPK (reads per thousand sequence reads), was proposed (see Supporting Information for detail). We first assessed the performance of nanopore sequencing using 845 samples collected from Shanghai East Hospital. For pathogen detection, we used two reference standards: (1) a clinical culture gold standard and (2) a composite standard incorporated additional results from qPCR. We randomly divided the samples into a training set (*n* = 199 samples) and a validation set (*n* = 646 samples). Receiver operator characteristic (ROC) curves were developed at varying RPK values for the training set using the two standards (Figure 1A,B). The ROC curve for training set was used to obtain the optimal thresholds that maximized the Youden index; then the scoring algorithm proposed by Gu^4^ was used to calculate sensitivity and specificity. In the validation set, using the urine culture standard, the sensitivity for bacterial and fungal detection was 89.8% and 96.3%, respectively; the specificity was 76.9% and 90.1%, respectively (Figure 1C). Using the composite standard, the sensitivity for bacterial and fungal detection was 98.4% and 98.2%, respectively; the specificity for bacterial and fungal detection was 85.4% and 98.1%, respectively (Figure 1C). In all 845 samples, the performance was equivalent to that in the validation set (Figure 1D). A total of 823 samples with clear clinical diagnostic information (758 UTIs and 65 non‐UTIs) were used for diagnostic performance assessment (Table S4). Nanopore sequencing obtained a significantly higher sensitivity for UTI diagnosis compared with urine culture (87.86% vs. 81.66%, *p* < .01), although the specificity was lower than that of urine culture (84.61% vs. 100%, *p* < .01) (Figure 1E,F). Among the 90 nanopore sequencing false negative samples, 80 samples were weakly positive (pathogen reads were detected but not reached the thresholds), and the remaining 10 samples had few microbial reads (Figure S2A, Table S5). Among 10 nanopore sequencing false‐positive samples, 6 samples were likely to reflect latent infections, because they came from patients with risk factors for UTI and clinical symptoms (Figure S2B). Furthermore, the detection frequency of mixed infection in UTI (with two or more organisms) of nanopore sequencing was much higher than that of urine culture (33.51% vs. 5.81%, *p* < .01) (Figure 1G and Table S6). Subanalysis showed that UTI diagnosis sensitivity and specificity of nanopore sequencing were higher in females compared to males (Figure S3).

**FIGURE 1:**
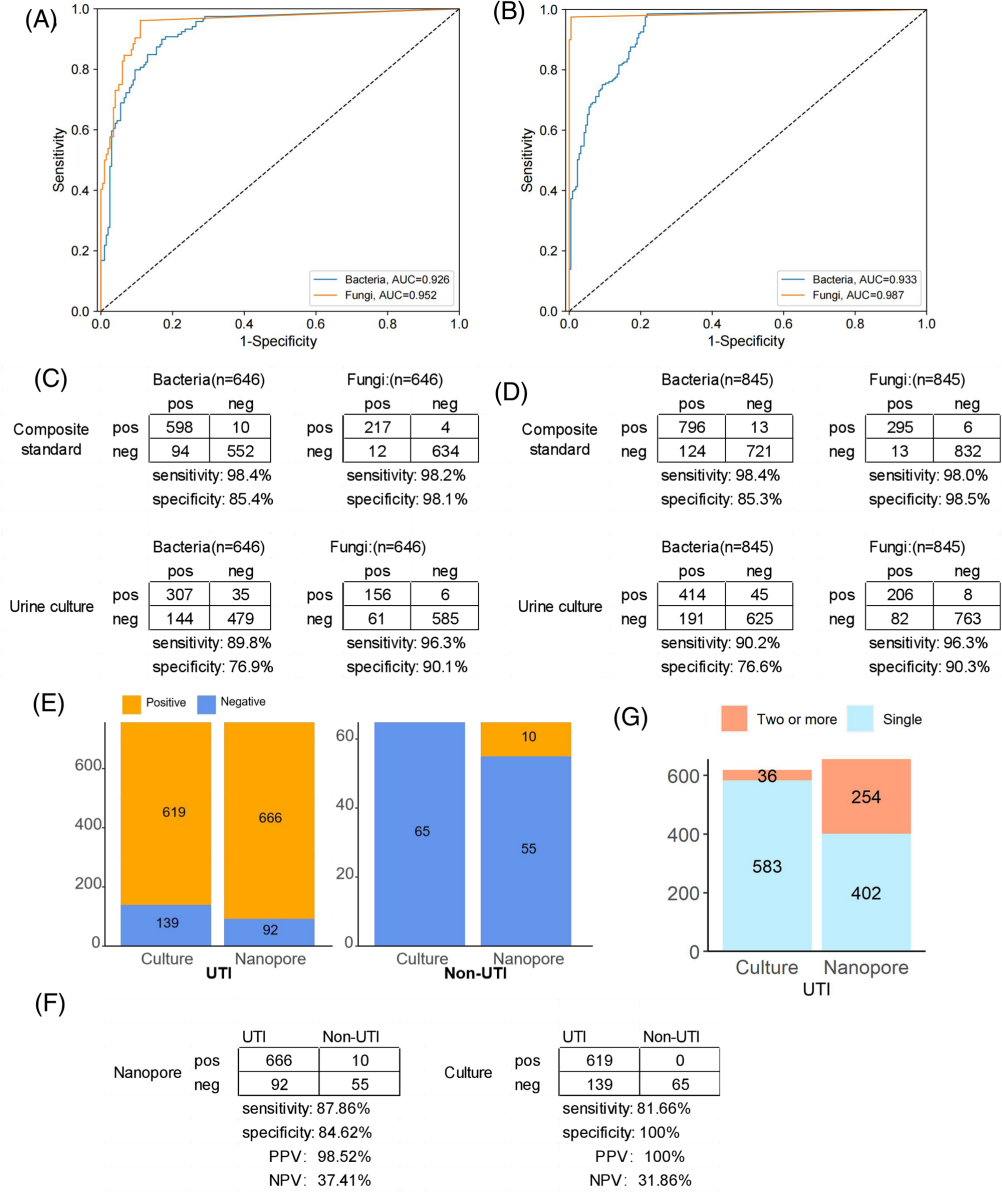
Pathogen detection and urinary tract infection (UTI) diagnosis performance of nanopore sequencing: (A) receiver operator characteristic (ROC) curves of training set for bacterial and fungal detections using clinical gold standard. Plots show nanopore‐sequencing test sensitivity and specificity, relative to the urine culture, at reads per thousand sequence reads (RPK) threshold values ranging from .1 to 1000; (B) ROC curves of training sets for bacterial and fungal detection using composite standard; (C) contingency tables for validation set; (D) contingency tables for all 845 samples; (E) comparison of metagenomics nanopore sequencing and urine culture detection results in UTI and non‐UTI groups; (F) contingency tables show the diagnostic performance of urine culture and nanopore sequencing for UTI and non‐UTI differentiating. PPV: positive predictive value; NPV: negative predictive value; pos: positive; neg: negative; (G) barplots show the distribution of samples with single organism or two or more organisms detected by culture and metagenomics nanopore sequencing.

Next, we validated the pathogen detection performance of metagenomics nanopore sequencing in other three hospitals. For bacteria detection, using composite standard, the sensitivity was 98.9%, 97.6% and 95.4%, respectively; the specificity was 68.3%, 79.7% and 76.3%, respectively (Figure 2). For fungal detection, using composite standard, the sensitivity was 100%, 100% and 85.7%, respectively; the specificity was 96.7%, 98.4% and 97.4%, respectively (Figure 2). The concordance between nanopore sequencing and urine culture in sample level is shown in Figure S4A,B. In samples with inconsistent nanopore sequencing and culture results, concordance between nanopore and qPCR is shown in Figure S4C.

**FIGURE 2:**
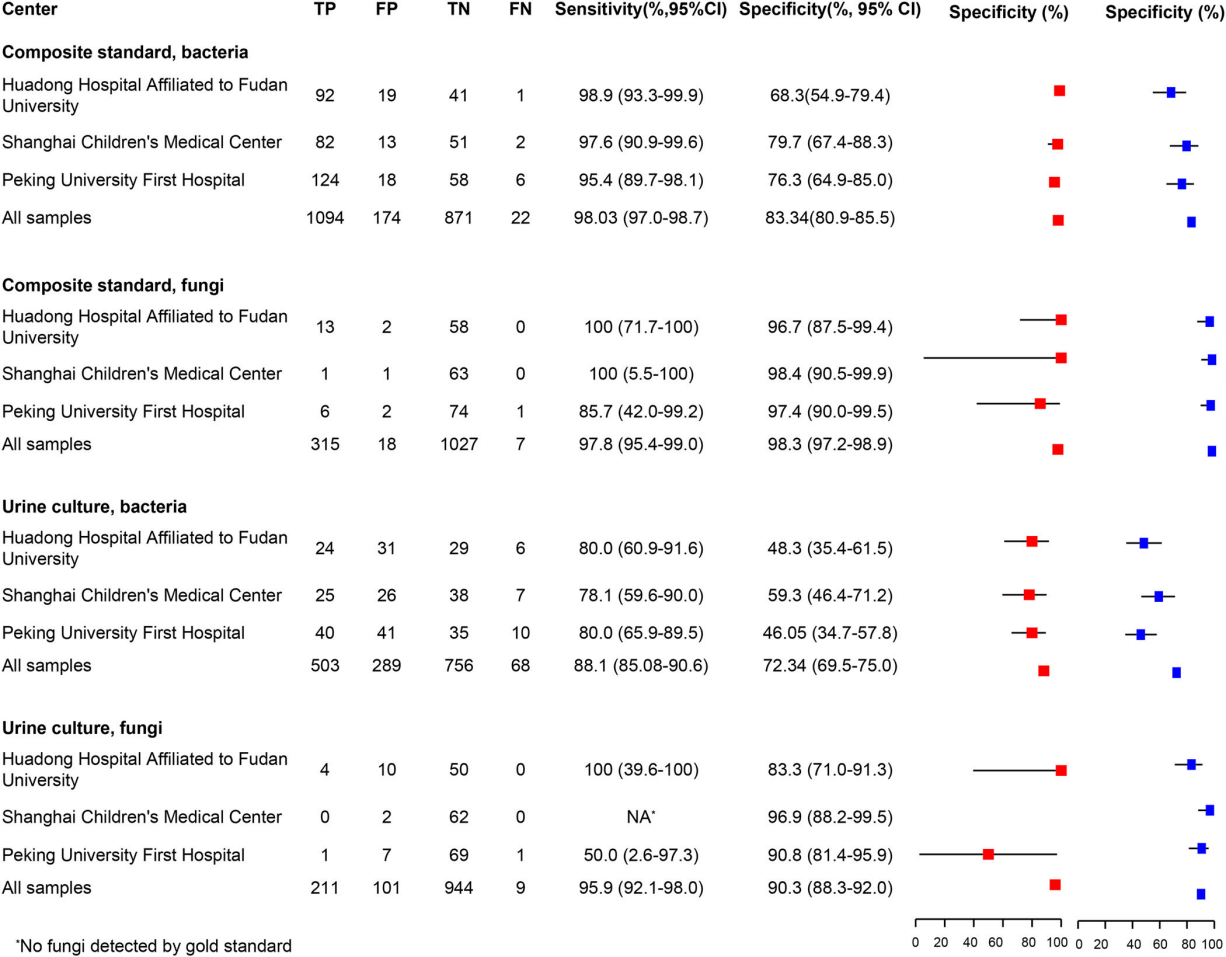
Test performance of metagenomics nanopore sequencing in three validation hospitals and all 1045 samples.

For comparison in pathogen level, as shown in Figure 3, nanopore sequencing improved the detection of common UTI pathogens, such as *Escherichia coli*, *Enterococcus faecium* and *Candida albicans*, as well as pathogens that are difficult to culture or rare in UTIs, such as *Enterococcus hirae* ^5^ and *Salmonella enterica*.^6^ The most commonly detected pathogens were generally consistent among the four centres and in agreement with previous studies^7^ (Figure S5).

**FIGURE 3:**
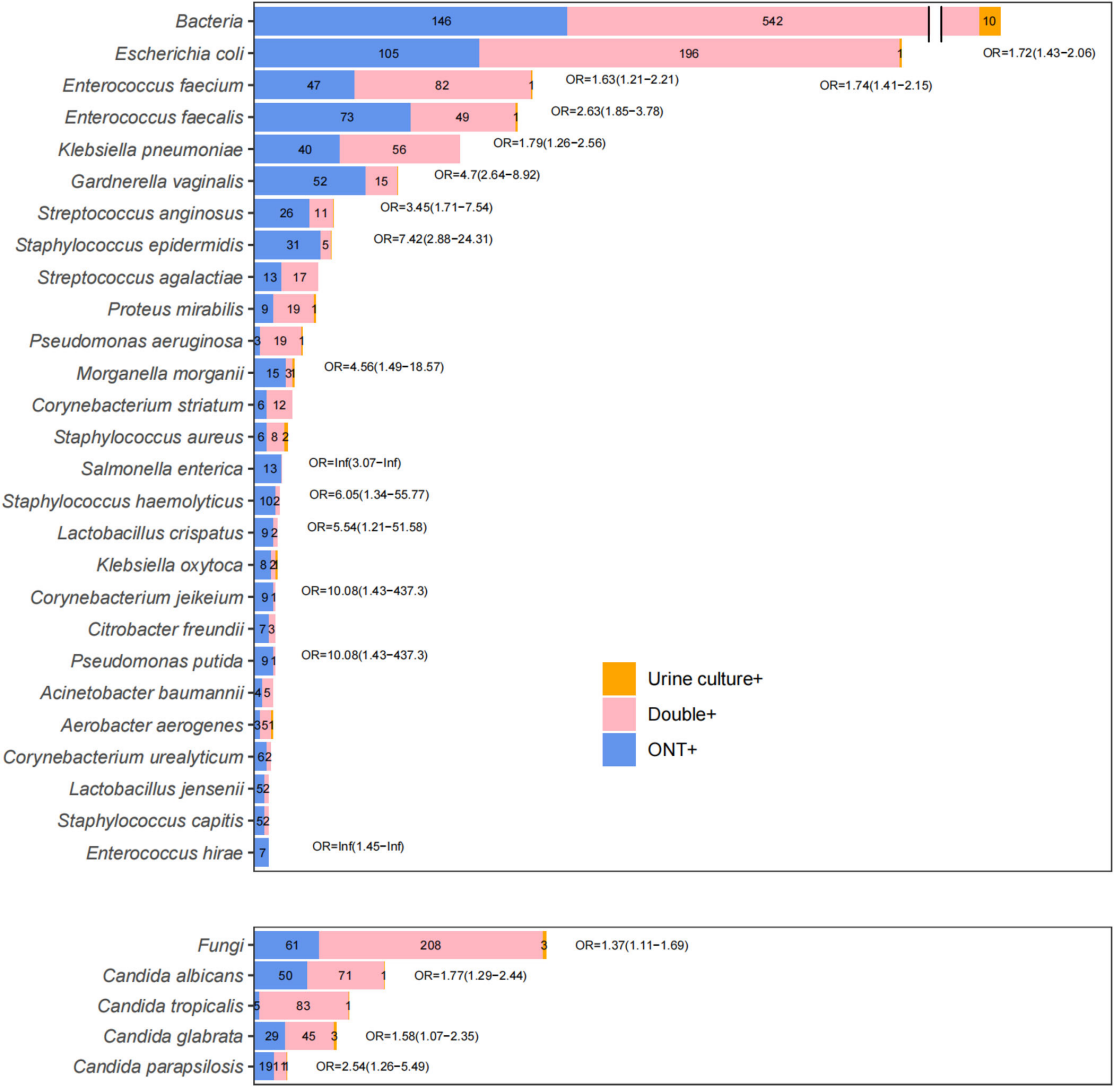
Comparison of nanopore sequencing and urine culture in terms of pathogens. The top 20 frequently detected bacteria and all fungi species detected are listed with their corresponding frequencies plotted in histograms. OR and *p* values from Fisher's exact test are shown for pathogens with significantly different detection rates between nanopore sequencing and urine culture.

We developed an in‐house pipeline “NanoAMR” to determine the exact AMR gene subtype. Testing using simulated data showed that when the genomic depth was above 20×; NanoAMR could detect AMR gene subtype accurately (Table S7). Then we performed AMR gene detection in samples with antibiotic susceptibility testing results available. In 239 of 317 samples showed resistance or mediation to at least one type of drug we tested; corresponding resistance genes were detected by nanopore sequencing (Table S8). The incidence of UTIs caused by *Enterobacterales* species, with extended‐spectrum beta‐lactamase (ESBL)‐positive or carbapenem‐resistant (CR) phenotype is increasing and poses a heavy burden on the hospital environment.^8^, ^9^ Therefore, we then focused on the detection of genes related to carbapenem resistance and ESBL‐positive phenotype in *E. coli* and *Klebsiella pneumoniae* (Figure 4). For *E. coli*, nanopore sequencing detected *bla*_NDM_ genes in two of three urine samples exhibiting the CR phenotype. Nanopore sequencing detected *bla*_CTX‐M_ genes in 37 of the 43 samples exhibiting ESBL‐positive phenotype, most commonly *bla*_CTX‐M‐14_. For *K. pneumoniae*, in agreement with the phenotypes, *bla*_KPC_ genes were detected in the six urine samples exhibiting serine enzyme–producing phenotype, and *bla*_NDM‐5_ was detected in the one urine sample exhibiting MBLs‐producing phenotype. *Bla*_SHV_ or *bla*_CTX‐M_ was detected by nanopore sequencing in all five urine samples exhibiting ESBL‐positive phenotype (Figure 4). These results showed that nanopore sequencing could detect the corresponding AMR genes for *E. coli* and *K. pneumoniae* exhibiting CR‐ or ESBL‐producing phenotype with high sensitivity, especially for *K. pneumoniae*, the sensitivity reached 100% in 12 samples (Tables S9 and S10).

**FIGURE 4:**
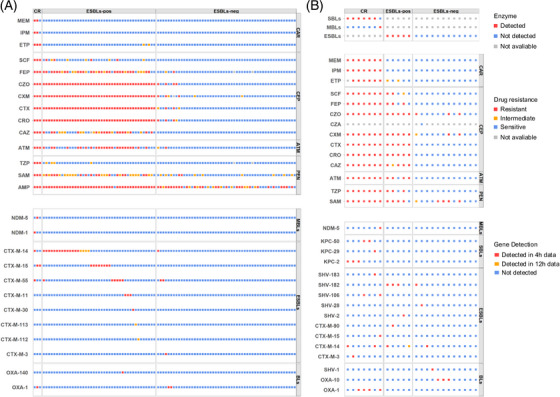
Detection of beta‐lactamase genes related to carbapenem resistance and extended‐spectrum beta‐lactamase (ESBL)‐positive phenotype in *Escherichia coli* (A) and *Klebsiella pneumoniae* (B): (A) top panel, results of antibiotic susceptibility tests for cultivated *E. coli* isolates; bottom panel, beta‐lactamase genes distribution in carbapenem‐resistant (CR), ESBL‐positive (ESBL‐pos) and ESBL‐negative (ESBL‐neg) *E. coli* groups; (B) top panel, enzyme detection results for cultivated *K. pneumoniae* isolates; middle panel, results of antibiotic susceptibility tests for cultivated *K. pneumoniae* isolates; bottom panel, beta‐lactamase genes distribution in CR, ESBL‐positive (ESBL‐pos) and ESBLs‐negative (ESBL‐neg) *K. pneumoniae* groups. (A and B) IPM, imipenem; MEM, meropenem; FEP, cefepime; ETP, ertapenem; SCF, cefperazone–sulbactam; CAZ, ceftazidime; CTX, cefotaxime; CRO, ceftriaxone; CXM, cefuroxime; CZO, cephazolin; ATM, aztreonam; TZP, piperacillin–tazobactam; SAM, ampicillin‐sulbactam; AMP, ampicillin; PEN, penicillin; CEP, cephalosporin; CAR, carbapenem.

In summary, we optimized metagenomics nanopore sequencing and bioinformatics pipeline suitable for detecting pathogens and AMR genes directly from urine samples within 10 h and assessed the performance of the methods in large‐scale samples. Notably, in‐house bioinformatics pipelines enable AMR gene detection with high sensitivity directly from urine, thus enabling clinicians to adjust antimicrobial therapy in a timely manner.

## CONFLICT OF INTEREST STATEMENT

The authors declare no potential conflict of interest.

## Supporting information

Supporting Information

Supporting Information

Supporting Information

Supporting Information

Supporting Information

Supporting Information

Supporting Information

Supporting Information

Supporting Information

Supporting Information

## Data Availability

The FASTQ data for this study are available from the corresponding author upon reasonable request. Codes for bioinformatics analysis are available in https://github.com/simceredx22/simNanoUTI.
