# Supplementary material for: Detection of pathogens and antimicrobial resistance genes directly from urine samples in patients suspected of urinary tract infection by metagenomics nanopore sequencing: A large‐scale multi‐centre study
Source: Clin Transl Med. 2023 Apr 26;13(4):e824. doi: 10.1002/ctm2.824 (PMC10131482; doi:10.1002/ctm2.824)
Supplement: Supplementary file 1 — Supporting Information [file CTM2-13-e824-s001.docx]

## Sample collection and storage

A total of 1063 patients with suspected UTI from four hospitals—Shanghai East Hospital, Shanghai Children's Medical Center, Huadong Hospital Affiliated with Fudan University and Peking University First Hospital—between June 2019 and April 2021 were screened. After exclusion of 18 patients with contaminated samples, 1,045 patients were enrolled in the study. The diagnosis of UTI was performed according to the Guidelines for Diagnosis and Treatment of Urological Diseases in China (2019 Edition).Specifically, patients with suspected UTI with symptoms of frequent, urgent or painful urination were diagnosed with UTI if they had abnormal urinalysis results or positive urine cultures. Spot, morning and midstream urine samples (5–30 mL) were collected and stored at 4°C for subsequent analysis. The study was approved by the ethics committee of Shanghai East Hospital (No.2018-035) and was conducted in accordance with the Declaration of Helsinki (as revised in 2013). Written informed consent was obtained from all patients or their close relatives. Their personal information is not disclosed publicly.

## Sample processing, DNA extraction and host depletion

Samples were treated using an optimized version of a previously reported sample preparation protocol ^1^. Each urine sample was centrifuged at 12300 rpm for 5 min, the supernatant was removed, and the pellet was resuspended in 1 mL PBS (Sangon Biotech). For host DNA depletion, the mix from the previous step was centrifuged at high speed to generate a pellet and supernatant; the supernatant was carefully discarded, and the pellet was fully resuspended in 250 μL of PBS. Subsequently, 200 μL of saponin was added, mixed well and incubated at room temperature on a rotary mixer. After incubation, 350 μL of nuclease-free water was added and vortex-mixed for 30 s, and 12 μL of 5 M NaCl was added and shaken immediately. Samples were then centrifuged, the supernatant was removed, and the pellet was resuspended in 100 µL of PBS. An additional 100 µL of HL-SAN buffer was added with 1 μL of HL-SAN DNase and incubated at 37°C for 10 min. The host DNA depleted samples were centrifuged, the supernatant was removed, and the pellet was resuspended in 800 μL of PBS. The samples were mixed by pipette and then transferred to FastPrep tubes. The bacterial cell walls were disrupted with a FastPrep cell homogenizer (MP Biomedicals) according to the manufacturer’s instructions. Lysates were high-speed centrifuged, and the supernatant was retained for genomic DNA extraction. DNA was then extracted with a DNeasy Blood & Tissue Kit (Qiagen, 69504) according to the manufacturer’s instructions. DNA quantification was performed with a Quant-it high-sensitivity dsDNA assay kit (Invitrogen) on a Qubit 3.0 Fluorometer (Thermo Fisher, Q33216).

## Library preparation and nanopore sequencing

DNA libraries were prepared with a Rapid PCR Barcoding Kit (SQK-RPB004, Oxford Nanopore Technologies) according to the manufacturer’s protocol. The PCR program used the following conditions: 97°C for 3 min initial denaturation, 35 cycles at 97°C denaturation for 20 s, 56°C annealing for 15 s, 65°C extension for 6 min and a final extension step at 65°C for 6 min.

Amplified DNA libraries were then sequenced with R9.4.1 flow cells on the GridION X5 platform (Oxford Nanopore Technologies) according to the manufacturer’s instructions. On average, 240 M of data were generated for each sample. ONT MinKNOW software (Version 4.3.1) was used to collect raw sequencing data and for local base-calling of the raw data (fast5 files) into reads (fastq files).

## Sequencing data pre-processing and pathogens database

Raw fast5 files were basecalled using HAC model with MinKNOW software v.4.3.1 installed on the GridION in real-time mode. For sequencing data quality control, we filtered out reads shorter than 500 bp and those with low quality (mean q-score <8). Subsequently, human reads were removed with minimap2 ^2^ to align to the human GRCh38.p13 genome and YanHuang genome. We built a SimMicro database for pathogen identification, as explained detailed below.

1. Sequences of bacteria, fungi, viruses and parasites were collected from the NT database.
2. Sequences that were too short (less than 1000 bp for bacteria/fungi, or less than 500 bp for viruses) and plasmid engineering vectors or bacteriophages sequences were removed, genome sequences for pathogenic bacteria and fungi from the refseq database were added to the database.
3. When genome sequences of multiple strains of the same species existed, we included as many strains as possible with genomes with high quality, accurate annotation and completeness.

In total, the SimMicro database included 10521 bacterial, 571 fungal, 8515 viral and 311 parasite reference genomes.

## Comparison with NGS

We sequenced a total of 61 samples with both GrindonX5 and the Illumina platform. With urine culture as the gold standard, Illumina sequencing returned more false positive results than nanopore sequencing (Figure S6).

## Limits of detection

To determine the LoDs for *Staphylococcus aureus*, *Escherichia coli* and *Candida albicans*, which are common pathogens in UTIs, we prepared mock samples consisting of these three pathogens spiked-into negative urine at concentrations of 4,900, 8,800 and 10,700 CFU/mL, respectively. The samples were serially diluted 1:10 four times, and three replicates were tested at each concentration. The LoD was defined as the lowest concentration at which the spiked-in pathogens were detected in all three replicates.

## Precision

We used mock samples consisting of *Staphylococcus aureus*, *Escherichia coli*, *Streptococcus agalactiae* and *Candida albicans* spiked into negative urine samples at concentrations of 1.0e + 04 CFU/ml to determine the precision of the method. For these samples, two experimental operators repeated the detection five times each to evaluate the precision between batches.

## Urine culture, antibiotic susceptibility testing and urinalysis

All midstream urine samples were inoculated on Columbia blood agar plates, MacConkey agar No.3 and Sabouraud dextrose agar plates and incubated in a carbon dioxide incubator at 35°C. Bacterial isolates were identified with MALDI-TOF MS (Autof ms1000). Antibiotic susceptibility testing was performed with a Vitek 2 compact system (bioMérieux, France) or the disk diffusion method as recommended. The control strain was *Escherichia coli* ATCC 25922. Susceptibility breakpoints were interpreted according to the Clinical and Laboratory Standards Institute guidelines ((CLSI) M100-S30 (2020)). Urinalysis tests were measured with a SYSMEX UF-1000i fully automatic urine analyzer according to the routine protocols in the hospital clinical laboratory.

## qPCR assays

qPCR was performed with a LightCycler 480 Instrument (Roche) to verify the inconsistent pathogen identification results between nanopore sequencing and urine culture. Primers were designed according to general rules and were used for qPCR verification (Table S11). SYBR-Green- and probe-based qPCR were performed on specific pathogen DNA fragments. SYBR-Green-based qPCR reactions were performed with 0.3 µL each of the reverse and forward primer (final concentration 0.2 μM), tagmented DNA (2 μl), 7.5 µL AceQ qPCR SYBR Green Master Mix (Vazyme) and nuclease-free water in a total volume of 15 µL. Probe-based qPCR reactions were performed with 0.3 µL each of the reverse and forward primer (final concentration 0.2 µM), tagmented DNA (2 μL), 7.5 µL TaqMan buffer (Invitrogen) and nuclease-free water in a total volume of 15 µL. SYBR-Green-based qPCR was used with the following conditions: pre-incubation at 95°C for 5 min, and 45 cycles at 95°C denaturation for 30 s, 60°C annealing for 15 s and 72°C extension for 30 s. Probe-based qPCR was used with the following conditions: pre-incubation at 95°C for 20 s, and 40 cycles at 95°C for 3 s and 60°C for 30 s.

## Method for pathogen identification

We developed a pathogen identification method to assess and optimize performance accuracy. The method included filtering out taxonomically related microorganisms, qPCR validation, calculating an RPK pathogen count and defining criteria for pathogen detection, as explained in detail below.

1. Filtering out misalignment caused by closely related microorganisms. Taxonomic classification with metagenomic data commonly yields a minor fraction of reads that map to related taxa with the same family or genus as that of a microorganism truly present in the sample. In our study, a filtering system based on “second best match reads ratio” was proposed. The steps are as follows. (i) reads were blasted to the SimMicro database, for each read, only the blast results with bit score higher than 95% of the highest bit score were kept. (ii) reads were extracted, grouped by species with highest blast bit score. (iii) for each species, calculating the total number of reads grouped into the species, and recording it as N; calculating the number of reads from this group blasted to other species, among which the species with the largest number of reads blasted to was defined as second best match species, recording the number of reads blasted to second best match species as M. (iv) calculating M/N, if M/N ≥18% (Figure S7A), the blast results of the species which the reads was grouped into were removed from blast results. After that, reads are grouped by best matched species again and reads number for each best match species is calculated. For example, if 100 reads were blasted to *Shigella sonnei* with highest blast bit score, meanwhile, 30 of the 100 reads could be blasted to *E.coli*, then the blast result of *Shigella sonnei* will be removed from blast result. Testing using 114 samples showed that pathogens detection based on our in-house filtering system outperformed Centrifuge ^3^, which used unique mapped ratio to filtering out closely related microorganisms (Figure S7B).
2. qPCR validation. qPCR was performed when pathogens detected by nanopore sequencing were inconsistent with urine culture results.
3. Calculating an RPK. We used RPK (reads per thousand) to standardize microorganisms across samples with uneven sequencing depths. RPK was defined as the number of pathogen reads divided by the number of basecalled reads. ROC curves were plotted with the Python software package and pandas data analysis library. The optimal RPK threshold was obtained by plotting the ROC curves at varying RPK values and determining the RPK at Youden’s index.
4. Criteria for pathogen detection. We developed three criteria for pathogen detection. The candidate pathogen was required to (1) the second best match ratio <18%, (2) have a minimum number of pathogen-specific reads identified (Table S12, ≥200 for both bacteria and fungi) and (3) meet an optimal RPK threshold. Optimal RPK thresholds with composite standards were set to the maximum Youden’s index (RPK threshold of 1.224 and 0.6041 for bacteria and fungi, respectively), as determined from the ROC curve of the training set.

All remaining microorganisms were compared with our clinical reportable range list for a final round of filtering (Table S13). It should be noted that only species-level identification was sought, not strain-level identification.

## AMR genes analysis

Nanopore sequencing data at 4 hours were extracted, in fasta format, from raw HDF5 files. AMR genes analysis was performed using NanoAMR, a in-house bioinformatics pipeline including three steps, as explained in detail below.

1. Preparing a single nucleotide polymorphism matrix (SNPM). AMR gene sequences were downloaded from Comprehensive Antibiotic Resistance Database (CARD, version 3.1.2) ^4^. Then the sequences were clustered with a sequence identity threshold of 0.95, and a representative sequence for each cluster was returned by CD-HIT (version 4.8.1) ^5^. After that, sequences in the same cluster were mapped to the representative sequence using minimap2 (version 2.21) ^2^. Then SAMtools ^6^ (version 1.13) tview command was used to display alignment and mark locations with different bases among sequences. We called the output of SAMtools tview as SNPM.
2. Classifying reads into AMR gene clusters. Reads from one sample were mapped against CARD sequences using BLAST (version 2.10.1+). If a read mapped to multiple AMR gene subtypes, the subtype with highest mapping score was reserved. Then reads were classified into AMR gene clusters according to the AMR gene subtypes they mapped. It should be noticed that reads from one sample could be classified into one or more AMR gene clusters.
3. Derterming the AMR gene subtype. To determine the exact AMR gene subtype of each sample, for each cluster, the reads classified into the cluster were mapped against all AMR gene subtypes in the cluster using minimap2 ^2^. Then SAMtools ^6^ tview was used to display alignment. The mapping score for different subtypes was calculated limited to SNP sites in SNPM in a site-specific manner. For each read, the record with highest mapping score was retained. Then the gene subtype with most reads assigned to was reported.

Determination of AMR genes in *E. coli* and *K. pneumoniae* with CR and ESBLs-positive phenotypes was performed by determining the presence of 1350 AMR genes subtype belonging to the 18 beta-lactamase gene family (Table S14). This list was compiled on the basis of a combination of literature review and data from public databases. For samples with CR or ESBLs-positive phenotype while beta-lactamase genes were not detected, sequencing data at 12 hours were further analyzed.

## Performance evaluation of NanoAMR using simulation data

We used simulation data of *E.coli.* to test the performance of NanoAMR in accurately determining AMR gene subtype. The details were described as follows.

1. Complete genome of *E.coli* strains which were isolated from human were collected from seven studies (Table S7). Then genomes predicted to contain NDM-5, CTX-M-14, CTX-M-15, OXA-1 genes by AMRFinderPlus software were retained. After that, literature review was performed to filter out genomes that have inconsistent results of AMRFinderPlus prediction and literature reports. Finally, 18 genomes were used to generate simulation data.
2. NanoSim^7^ software was used to generate simulation data. For each genome, a total of 80,000 reads with a length between 2000-6000 bp were generated using predefined error profile ecoli_R9_1D ecoli_R9.4_1D.
3. For each simulation data sets, AMR genes were detected using NanoAMR in different genome depth. Then we counted the number of genomes detected the exact AMR gene subtype by NanoAMR and other AMR gene subtype from the AMR gene family by NanoAMR.

## Statistical methods

To evaluate the test accuracy of metagenomic nanopore sequencing for bacterial and fungal detection, we used two reference standards: (1) a clinical gold standard consisting of available cultures and (2) a composite standard that incorporated additional results from qPCR. According to Gu et al. ^8^, the specific scoring algorithm is outlined as follows (Table S15): on the basis of the clinical or composite standard, true positives or false negatives were scored for each microorganism detected or not detected by nanopore sequencing. It should be noted that nanopore sequencing for some samples detected additional pathogens compared with urine culture, but qPCR verification was not performed. We counted all these results as false positive, which may lead to an underestimation of specificity to a certain extent. For each sample, a true negative was scored if no other microorganism(s) other than those expected, on the basis of the clinical or composite standard, were detected by nanopore sequencing; otherwise, a false positive was scored. Multiple false-positive results in a sample were counted as one false positive overall. For sample level comparison, match means the detected species of two methods were exactly same, partly-matched means, at least one pathogens overlapped between two methods. In the definition of mixed infection, for species detected by nanopore, we only counted the species with positive culture or verified by qPCR. Data analyses were performed in R 4.0.3 software. Comparative analysis was conducted with Fisher’s exact test. p values <0.05 were considered significant, and all tests were two-tailed.

1. Charalampous T., Kay G. L., Richardson H., Aydin A., Baldan R., Jeanes C., Rae D., Grundy S., Turner D. J., Wain J., Leggett R. M., Livermore D. M., O'Grady J. Nanopore metagenomics enables rapid clinical diagnosis of bacterial lower respiratory infection. *Nat Biotechnol* 2019;37:783-792.

2. Li Heng. Minimap2: pairwise alignment for nucleotide sequences. *Bioinformatics* 2018;34:3094-3100.

3. Kim D., Song L., Breitwieser F. P., Salzberg S. L. Centrifuge: rapid and sensitive classification of metagenomic sequences. *Genome Res* 2016;26:1721-1729.

4. Alcock Brian P., Raphenya Amogelang R., Lau Tammy T. Y., Tsang Kara K., Bouchard Mégane, Edalatmand Arman, Huynh William, Nguyen Anna-Lisa V., Cheng Annie A., Liu Sihan, Min Sally Y., Miroshnichenko Anatoly, Tran Hiu-Ki, Werfalli Rafik E., Nasir Jalees A., Oloni Martins, Speicher David J., Florescu Alexandra, Singh Bhavya, Faltyn Mateusz, Hernandez-Koutoucheva Anastasia, Sharma Arjun N., Bordeleau Emily, Pawlowski Andrew C., Zubyk Haley L., Dooley Damion, Griffiths Emma, Maguire Finlay, Winsor Geoff L., Beiko Robert G., Brinkman Fiona S. L., Hsiao William W. L., Domselaar Gary V., McArthur Andrew G. CARD 2020: antibiotic resistome surveillance with the comprehensive antibiotic resistance database. *Nucleic Acids Research* 2020;48:D517-D525.

5. Fu Limin, Niu Beifang, Zhu Zhengwei, Wu Sitao, Li Weizhong. CD-HIT: accelerated for clustering the next-generation sequencing data. *Bioinformatics* 2012;28:3150-3152.

6. Li Heng, Handsaker Bob, Wysoker Alec, Fennell Tim, Ruan Jue, Homer Nils, Marth Gabor, Abecasis Goncalo, Durbin Richard, Genome Project Data Processing Subgroup. The Sequence Alignment/Map format and SAMtools. *Bioinformatics* 2009;25:2078-2079.

7. Yang C., Chu J., Warren R. L., Birol I. NanoSim: nanopore sequence read simulator based on statistical characterization. *Gigascience* 2017;6:1-6.

8. Gu W., Deng X., Lee M., Sucu Y. D., Arevalo S., Stryke D., Federman S., Gopez A., Reyes K., Zorn K., Sample H., Yu G., Ishpuniani G., Briggs B., Chow E. D., Berger A., Wilson M. R., Wang C., Hsu E., Miller S., DeRisi J. L., Chiu C. Y. Rapid pathogen detection by metagenomic next-generation sequencing of infected body fluids. *Nat Med* 2021;27:115-124.
