# Supplementary material for: Detection of pathogens and antimicrobial resistance genes directly from urine samples in patients suspected of urinary tract infection by metagenomics nanopore sequencing: A large‐scale multi‐centre study
Source: Clin Transl Med. 2023 Apr 26;13(4):e824. doi: 10.1002/ctm2.824 (PMC10131482; doi:10.1002/ctm2.824)
Supplement: Supplementary file 3 — Supporting Information [file CTM2-13-e824-s005.docx]

**Figure S1.** Study workflow.

**Figure S2.** Analysis of discordant samples. (A) Four culture positive only samples obtained from patients diagnosed with UTI, barplot showed disturbution of nanopore sequencing weak positive and not detected. (B) Ten nanopore sequencing positive only samples obtained from patients diagnosed with non-UTI, barplot showed disturbution of samples obtained from patients with UTI risk factor or not. (C) 51 nanopore positive samples obtained from patients diagnosed with UTI, barplot showed the distribution of pathogens detected by nanopore sequencing.

**Figure S3.** UTI diagnosis performance of nanopore sequencing and culture in females and males. (A) Comparison of metagenomic nanopore sequencing and urine culture detection results in UTI and non-UTI groups in females. (B) Comparison of metagenomic nanopore sequencing and urine culture detection results in UTI and non-UTI groups in males. (C) Contingency tables show the diagnostic performance of metagenomic urine culture and nanopore sequencing for UTI and non-UTI differentiating in females. (D) Contingency tables show the diagnostic performance of metagenomic urine culture and nanopore sequencing for UTI and non-UTI differentiating in males.

**Figure S4.** Comparison of nanopore sequencing and urine culture for pathogens detection. (A) Pie chart demonstrating the positivity distribution of nanopore sequencing and culture for all samples. (B) Pie chart demonstrating distribution of Double+ samples. (C) Barplot for comparison of nanopore sequencing and qPCR results in the Double+, partly-matched, ONT+, and Double+, mismatch groups.

**Figure S5.** Pathogens detected in four centers. (A) Shanghai East Hospital, for bacteria, only top 20 frequently detected species were showed. (B) Peking University First Hospital, for bacteria, only top 20 frequently detected species were showed. (C) Huadong Hospital Affiliated with Fudan University. (D) Shanghai Children’s Medical Center.

**Figure S6.** Comparison of nanopore sequencing and Illumina sequencing for pathogens detection in 61 samples. (A) Barplot shows number of negative and positive samples by nanopore or Illumina sequencing in urine culture negative samples. (B) Barplot shows number of negative and positive samples by nanopore or Illumina sequencing in urine culture positive samples.

**Figure S7. Methods to filtering out misalignment caused by closely related microorganisms.** (A) Second best match ratio and unique mapped ratio in true positive (TP) and false positive groups in 36 samples. (B) Performance of in-house methods and Centrifuge in 78 validation samples and 114 samples
