# Supplementary figures and images for: Detection of pathogens and antimicrobial resistance genes directly from urine samples in patients suspected of urinary tract infection by metagenomics nanopore sequencing: A large‐scale multi‐centre study
Source: Clin Transl Med. 2023 Apr 26;13(4):e824. doi: 10.1002/ctm2.824 (PMC10131482; doi:10.1002/ctm2.824)

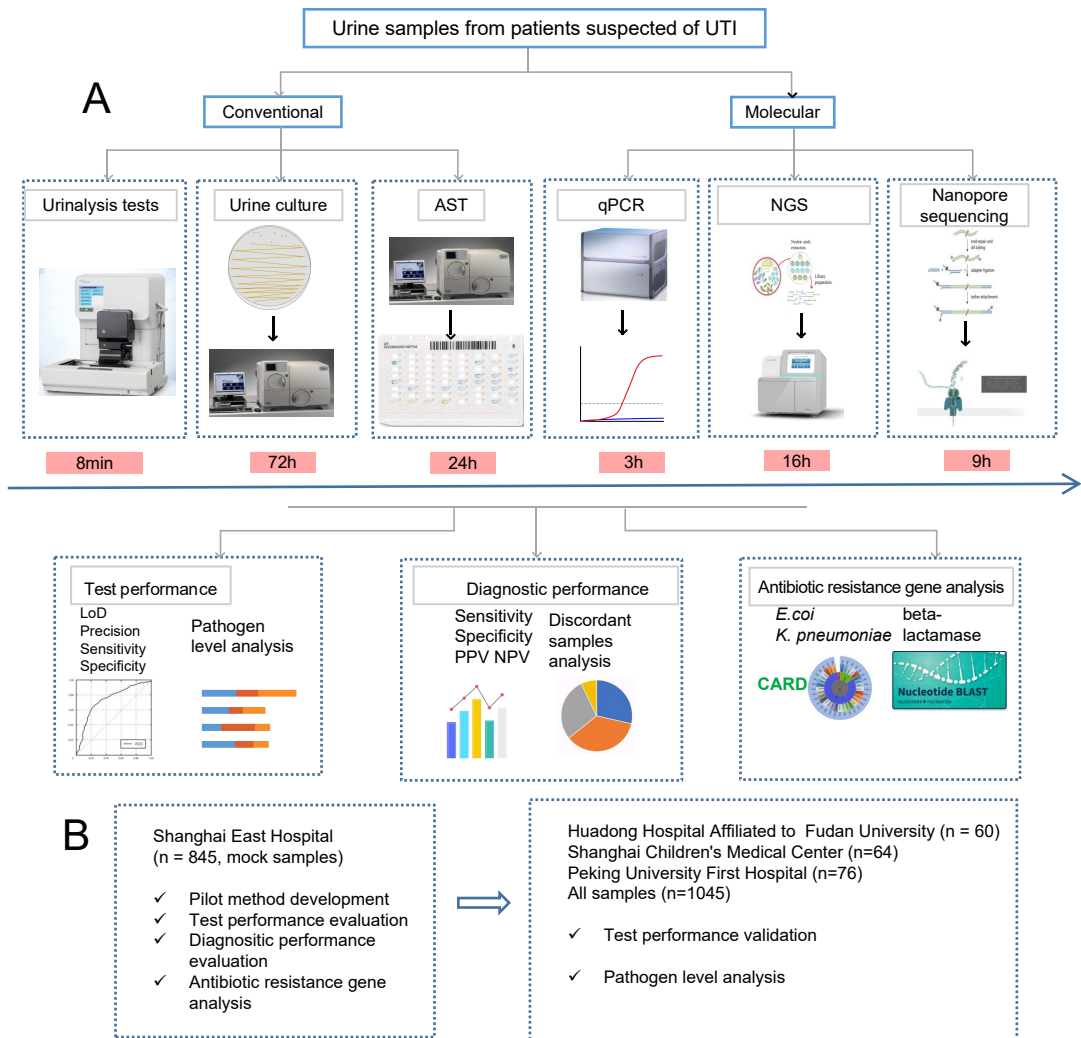

Supplement: Supplementary file 4 — Supporting Information [file CTM2-13-e824-s010.pdf]

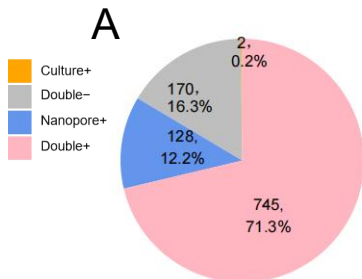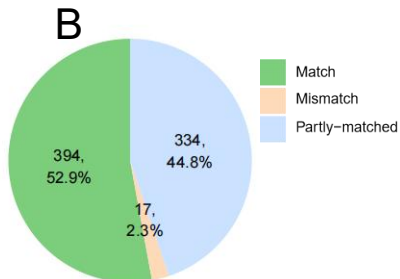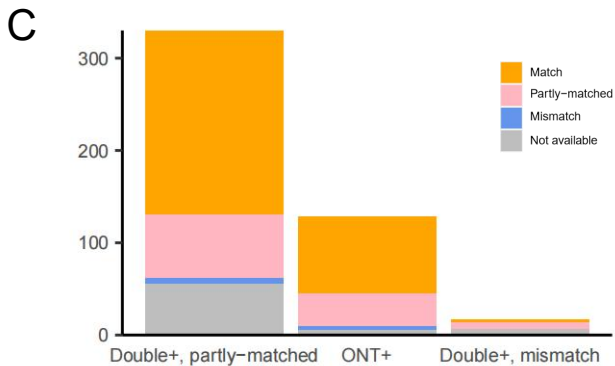

Supplement: Supplementary file 7 — Supporting Information [file CTM2-13-e824-s009.pdf]

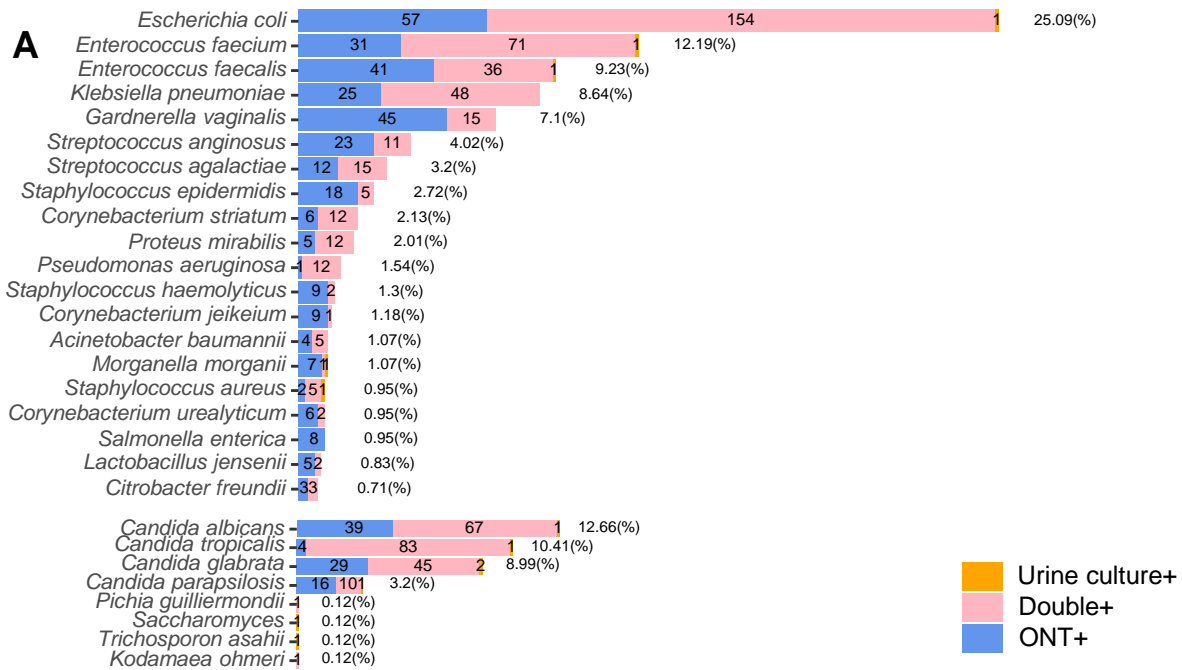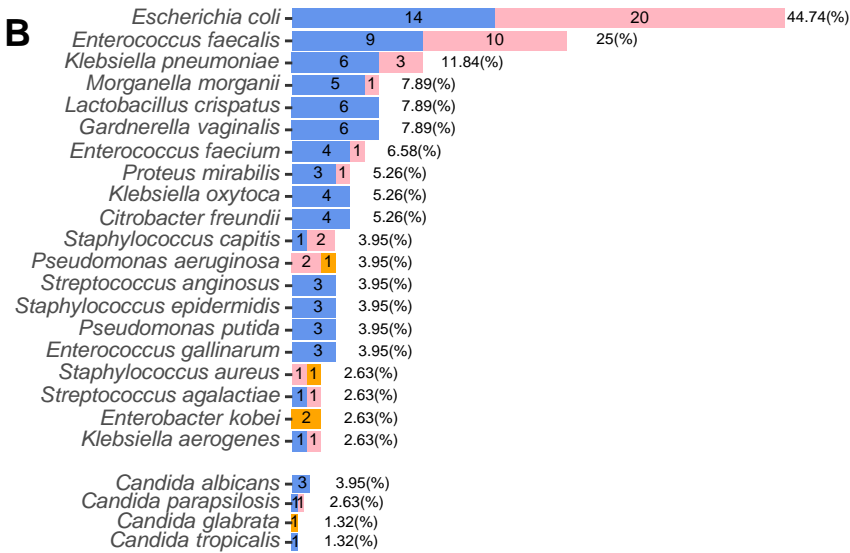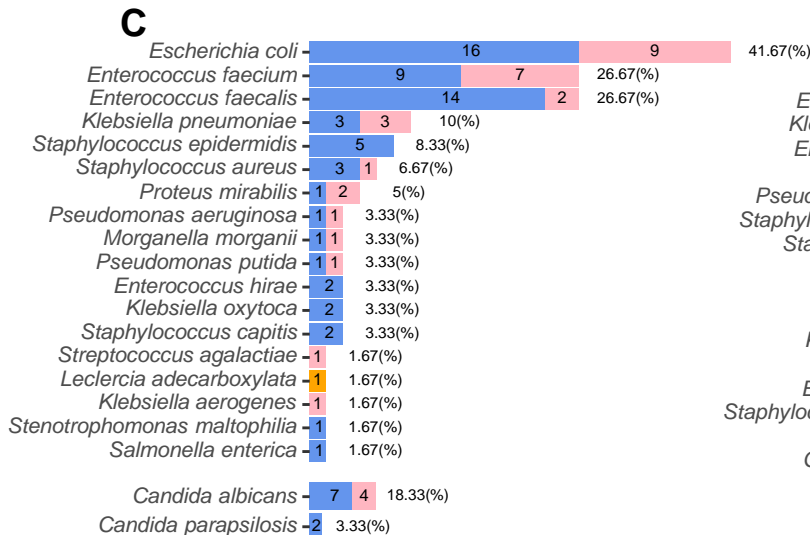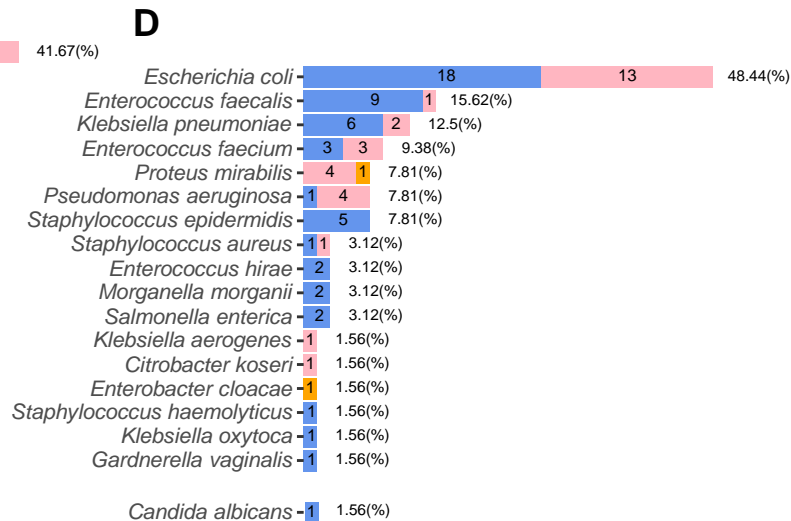

Supplement: Supplementary file 8 — Supporting Information [file CTM2-13-e824-s006.pdf]

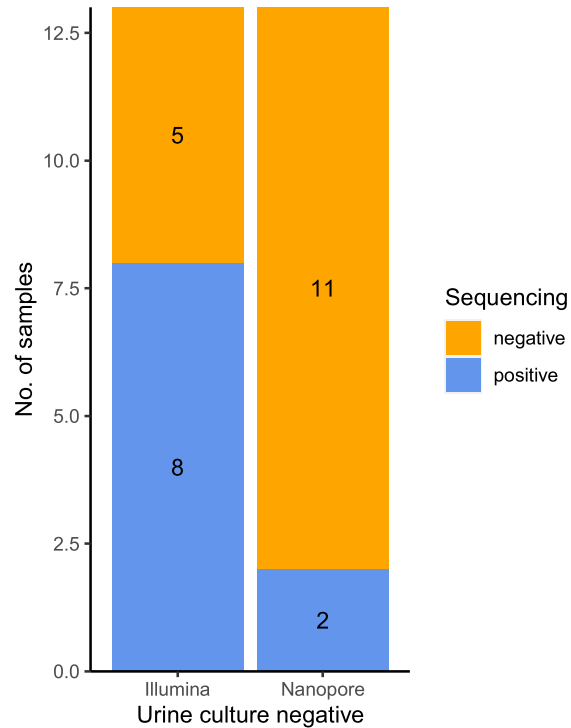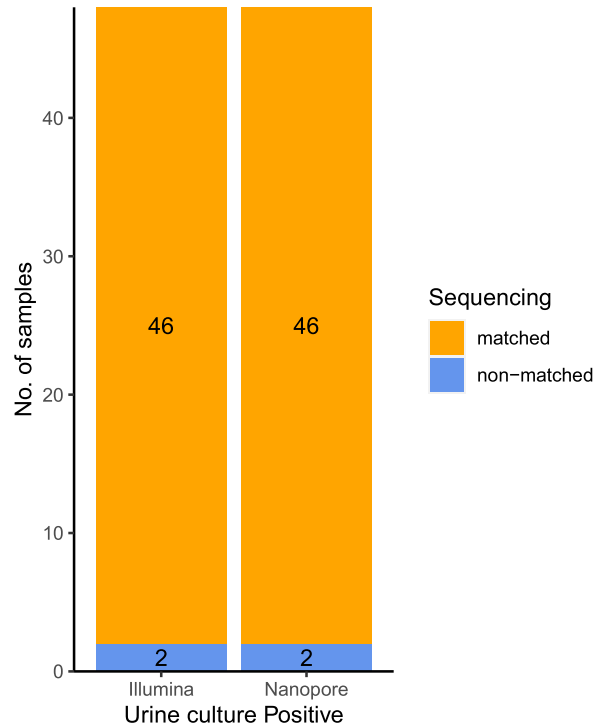

Supplement: Supplementary file 9 — Supporting Information [file CTM2-13-e824-s003.pdf]
