# Supplementary material for: Detection of pathogens and antimicrobial resistance genes directly from urine samples in patients suspected of urinary tract infection by metagenomics nanopore sequencing: A large‐scale multi‐centre study
Source: Clin Transl Med. 2023 Apr 26;13(4):e824. doi: 10.1002/ctm2.824 (PMC10131482; doi:10.1002/ctm2.824)
Supplement: Supplementary file 5 — Supporting Information [file CTM2-13-e824-s008.pdf]

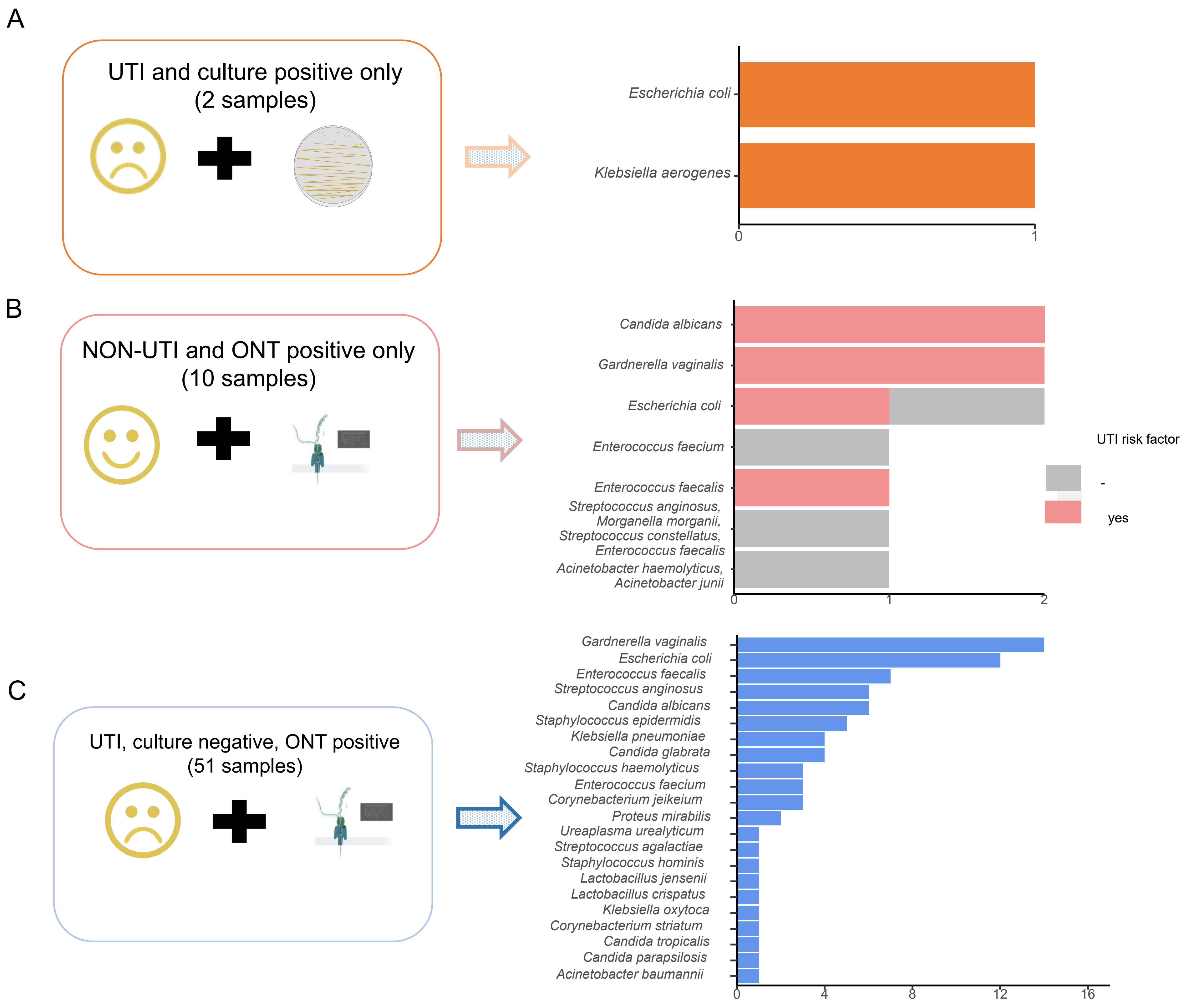
A
UTI and culture positive only
(2 samples)
Escherichia coli
Klebsiella aerogenes
0
1
B
NON-UTI and ONT positive only
(10 samples)
Candida albicans
Gardnerella vaginalis
Escherichia coli
Enterococcus faecium
Enterococcus faecalis
Streptococcus anginosus, Morganella morganii, Streptococcus constellatus, Enterococcus faecalis
Acinetobacter haemolyticus, Acinetobacter junii
0
1
2
UTI risk factor
-
yes
C
UTI, culture negative, ONT positive
(51 samples)
Gardnerella vaginalis
Escherichia coli
Enterococcus faecalis
Streptococcus anginosus
Candida albicans
Staphylococcus epidermidis
Klebsiella pneumoniae
Candida glabrata
Staphylococcus haemolyticus
Enterococcus faecium
Corynebacterium jeikeium
Proteus mirabilis
Ureaplasma urealyticum
Streptococcus agalactiae
Staphylococcus hominis
Lactobacillus jensenii
Lactobacillus crispatus
Klebsiella oxytoca
Corynebacterium striatum
Candida tropicalis
Candida parapsilosis
Acinetobacter baumannii
0
4
8
12
16
