# Supplementary material for: Detection of pathogens and antimicrobial resistance genes directly from urine samples in patients suspected of urinary tract infection by metagenomics nanopore sequencing: A large‐scale multi‐centre study
Source: Clin Transl Med. 2023 Apr 26;13(4):e824. doi: 10.1002/ctm2.824 (PMC10131482; doi:10.1002/ctm2.824)
Supplement: Supplementary file 6 — Supporting Information [file CTM2-13-e824-s007.pdf]

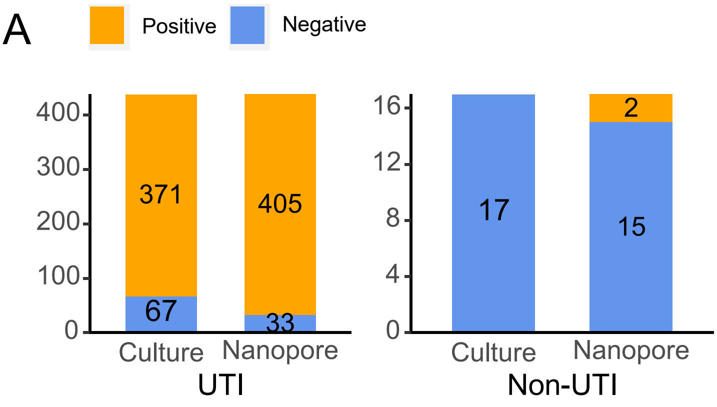

B

		UTI	Non-UTI
Nanopore	pos	405	2
	neg	33	15

sensitivity: 92.47%

specificity: 88.24%

		UTI	Non-UTI
Culture	pos	371	0
	neg	67	17

sensitivity: 84.70%

specificity: 100%

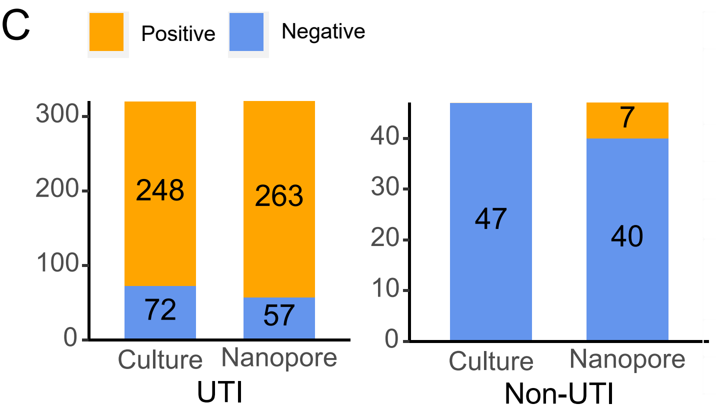

D

		UTI	Non-UTI
Nanopore	pos	263	7
	neg	57	40

sensitivity: 82.19%

specificity: 85.11%

		UTI	Non-UTI
Culture	pos	248	0
	neg	72	47

sensitivity: 77.50%

specificity: 100%
