# Supplementary material for: Detection of pathogens and antimicrobial resistance genes directly from urine samples in patients suspected of urinary tract infection by metagenomics nanopore sequencing: A large‐scale multi‐centre study
Source: Clin Transl Med. 2023 Apr 26;13(4):e824. doi: 10.1002/ctm2.824 (PMC10131482; doi:10.1002/ctm2.824)
Supplement: Supplementary file 10 — Supporting Information [file CTM2-13-e824-s002.pdf]

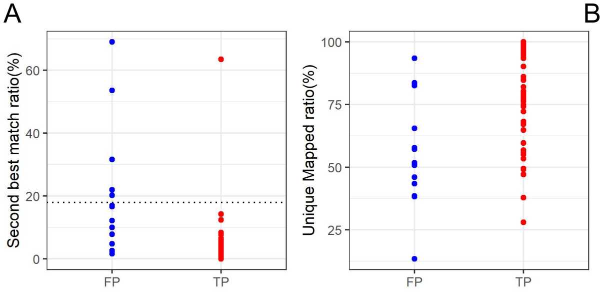

B

Validation dataset (n = 78)

Composite standard	in-house methods pos	in-house methods neg
pos	115	1
neg	31	47

sensitivity: 99.14%
specificity: 60.25%

Composite standard	Centrifuge pos	Centrifuge neg
pos	114	4
neg	38	40

sensitivity: 96.61%
specificity: 51.28%

All dataset (n = 114)

Composite standard	in-house methods pos	in-house methods neg
pos	185	1
neg	47	67

sensitivity: 99.46%
specificity: 58.77%

Composite standard	Centrifuge pos	Centrifuge neg
pos	181	4
neg	64	50

sensitivity: 97.84%
specificity: 43.86%
